# On the current status of *Phakopsora pachyrhizi* genome sequencing

**DOI:** 10.3389/fpls.2014.00377

**Published:** 2014-08-27

**Authors:** Marco Loehrer, Alexander Vogel, Bruno Huettel, Richard Reinhardt, Vladimir Benes, Sébastien Duplessis, Björn Usadel, Ulrich Schaffrath

**Affiliations:** ^1^Department of Plant Physiology, Rheinisch-Westfälische Technische Hochschule Aachen UniversityAachen, Germany; ^2^Institute for Botany and Molecular Genetics, Institute for Biology I, Rheinisch-Westfälische Technische Hochschule Aachen UniversityAachen, Germany; ^3^Max Planck Institute for Plant Breeding Research, KölnGermany; ^4^Genomics Core Facility, European Molecular Biology LaboratoryHeidelberg, Germany; ^5^Institut National de la Recherche Agronomique, Interactions Arbres/Microorganismes, UMR 1136, ChampenouxFrance; ^6^Université de Lorraine, Interactions Arbres/Microorganismes, UMR 1136, Vandoeuvre-lès-NancyFrance; ^7^Institute of Bio- and Geosciences-2 Plant Sciences, Institute for Bio- and Geosciences, Forschungszentrum Jülich, JülichGermany

**Keywords:** fungal genomics, rust fungi, Asian soybean rust, next-generation sequencing, herterozygosity, genome size, k-mer analysis

## Abstract

Recent advances in the field of sequencing technologies and bioinformatics allow a more rapid access to genomes of non-model organisms at sinking costs. Accordingly, draft genomes of several economically important cereal rust fungi have been released in the last 3 years. Aside from the very recent flax rust and poplar rust draft assemblies there are no genomic data available for other dicot-infecting rust fungi. In this article we outline rust fungus sequencing efforts and comment on the current status of *Phakopsora pachyrhizi* (Asian soybean rust) genome sequencing.

Sequencing of fungal genomes represented a significant milestone in the emerging era of “genomics.” In fact, the first eukaryotic genome ever sequenced was that of baker’s yeast, *Saccharomyces cerevisiae*, which consequently strengthened its position as a fungal model organism after the release of the 12 Mb genome with approximately 6000 genes in 1996 (Goffeau et al., 1996). Some time thereafter the genomes of the fission yeast *S. pombe* (14 Mb) and the filamentous ascomycete *Neurospora crassa* (40 Mb) were released in Wood et al. (2002) and Galagan et al. (2003), respectively. Accelerated progress in sequencing technology from early clone-by-clone approaches through Sanger-based whole-genome shotgun sequencing (WGS) to today’s next-generation sequencing (NGS) shortened the periods between releases of novel genomes considerably (Grigoriev, 2014). This paved the way for comparative genomics which opened new possibilities for people working in the field of agriculture and biotechnology or combating human, animal or plant diseases (Vebø et al., 2009; Manning et al., 2013; Bolger et al., 2014).

In the latter field, the sequencing of the genome of the ascomycete *Magnaporthe oryzae* was achieved by Dean et al. (2005). Along with the genome of rice (Goff et al., 2002), the *M. oryzae* host plant, an understanding of the plant–pathogen interaction became possible at the genome level. Since then, several plant-pathogenic fungi were sequenced; however, a group of pathogens that exclusively feed from living plant tissue, so-called obligate biotrophs, remained recalcitrant. This was disappointing particularly because some of the most economically serious threats to human nutrition, such as powdery mildew fungi and rust fungi, are among this group.

Rust fungi have long been in the focus of plant pathologists. Already in the 19th century, Anton de Bary, who is considered as a founder of plant pathology, picked up *Puccinia graminis* with its various *formae speciales* that are specialized for parasitism on particular cereal hosts, as subject for his groundbreaking studies. Later Harold Henry Flor developed the famous “gene-for-gene” concept based on his work on the interaction of flax rust (*Melampsora lini*) with its host plant flax (*Linum usitatissimum*; Flor, 1955). Despite considerable interest, sequencing of rust genomes was not achieved until most recently. Thus, the 101 Mb genome of *Melampsora larici-populina* and the 89 Mb draft genome of *Puccinia graminis* f. sp. *tritici* were sequenced in a common effort by the Joint Genome Institute and the Broad Institute, respectively, and published in Duplessis et al. (2011). Following, more or less advanced draft genomes of other rust fungi were sequenced and published by the community, such as several *Puccinia striiformis* f. sp. *tritici* races (56–110 Mb) and the flax rust genome *M. lini* (Cantu et al., 2011, 2013; Zheng et al., 2013; Nemri et al., 2014; see **Table 1**). Although Pucciniales is an order with a lesser coverage compared to other fungi^1^, more genomic resources are becoming accessible. A major drawback encountered during sequencing efforts of rust genomes was their unexpected large sizes, a fact that also hampered attempts of sequencing the genome of the Asian soybean rust fungus *Phakopsora pachyrhizi*, an economically important threat to soybean cultivation. The following commentary is written to give an overview on the current status of *P. pachyrhizi* genome sequencing and is intended to initiate combined activities toward this goal.

**Table 1:** Published rust fungi draft genomes and genome size estimations in alphabetical order.

Organism	Genome size (Mb)	Estimation/sequencing method	Reference
*Cronartium quercuum* f. sp. *fusiforme*	90	Flow cytometry	Anderson et al. (2010)
*Hemileia vastatrix*	733.5	Flow cytometry	Carvalho et al. (2013)
*Melampsora larici-populina*	101	Sanger sequencing	Duplessis et al. (2011)
*Melampsora lini*	189	Next-generation sequencing	Nemri et al. (2014)
*Puccinia coronata*	77	Flow cytometry	Eilam et al. (1994)
*Puccinia graminis* f. sp. *tritici*	89	Sanger sequencing	Duplessis et al. (2011)
*Puccinia hordei*	121	Flow cytometry	Eilam et al. (1994)
*Puccinia recondita*	127	Flow cytometry	Eilam et al. (1994)
*Puccinia sorghi*	102	Flow cytometry	Eilam et al. (1994)
*Puccinia striiformis* f. sp. *tritici*, race PST-130	65	Next-generation sequencing	Cantu et al. (2011)
*Puccinia striiformis* f. sp. *tritici*, races PST-21, PST-43, PST-87/7, PST-08/21	73, 71, 53, 56	Next-generation sequencing	Cantu et al. (2013)
*Puccinia striiformis* f. sp. *tritici*, isolate CY32	110	Next-generation sequencing, “fosmid-to-fosmid” sequencing	Zheng et al. (2013)
*Puccinia triticiana* 1-1 BBDB race 1	135	Assembly	Fellers et al. (2013)
*Uromyces appendiculatus*	418	Flow cytometry	Eilam et al. (1994)
*Uromyces vignae*	407	Flow cytometry	Eilam et al. (1994)

What makes *P. pachyrhizi* so interesting? For sure it is a devastating fungal disease of the important crop plant soybean. The origin of the pathogen can be traced back to Asia and most likely it spread alongside with the propagation of soybean cultivation. *P. pachyrhizi* is able to infect more than 31 species from 17 genera of legumes, which is a rather unusual feature for rust fungi that usually are highly specialized for particular hosts (Goellner et al., 2010). *P. pachyrhizi* differs in a further important aspect from the majority of rusts: it directly penetrates leaf cells rather than entering the leaf via stomata at the uredinial stage. On the contrary, most rust fungi use stomata to get inside the host tissues at this stage and a direct penetration is only observed for some rust fungi when basidiospores infect the aecial host at later stages of the rust life cycle (Heath, 1997). Recent studies imply that generation of high turgor pressure of around 5 MPa in the non-melanized appressoria supports penetration (Loehrer et al., 2014). Penetrated epidermal cells undergo a cell death response, again an unexpected property for a biotrophic pathogen. Experiments with non-host plants such as barley and Arabidopsis showed that during penetration and concomitant epidermal cell death, marker genes associated with responses to necrotrophic pathogens are switched on and that cell death suppression had a negative influence on infection success of *P. pachyrhizi* (Loehrer et al., 2008; Hoefle et al., 2009). Regarding its lifestyle, *P. pachyrhizi* which forms so far only a single spore type in the wild, i.e. urediospores, is a minimalist compared to, e.g., *Puccinia graminis* f. sp. *tritici* which has five distinct spore types and performs a host jump (Leonard and Szabo, 2005). Despite the unknown or missing sexual life cycle the genetic diversity of *P. pachyrhizi* seems not to be impaired. This may be explained by parasexual nuclear recombination occurring between different isolates after germ tube fusion or hyphal anastomosis, a feature also reported for cereal rusts (Wang and McCallum, 2009; Vittal et al., 2012).

Public information about the *P. pachyrhizi* genome sequencing project is rare. In the DoE JGI Community Sequencing Program of 2004, a project was launched to sequence the genome *of P. pachyrhizi* (isolate Taiwan 72-1) based on a fosmid shotgun sequencing approach. The genome size prediction with 50 Mb at that time was much underestimated. The sequencing project has now a “permanent draft” status at the JGI^2^. Besides the recently released mitochondrial genome sequence (Stone et al., 2010), information on assembly attempts of the nuclear genome have not been published. The major drawback for progress in *P. pachyrhizi* genome sequencing seems to be its huge size. An update on this topic was given at the National Soybean Rust Symposium 2005 in Nashville (TN, USA). Genome size estimations ranged from 300 to 950 Mb depending on the analysis method used (Posada-Buitrago et al., 2005). A similar statement was provided by Igor Grigoriev (Head of the JGI Fungal Program) suggesting a genome size above 850 Mb (Duplessis et al., 2012). Besides, other general features of rust fungi genomes unraveled since then, such as expanded multigene families and very large amount of transposable elements (>45%), pose serious problems for proper genome assembly.

We started our own efforts toward uncovering the genome size of *P. pachyrhizi* by using our lab isolate (Brazil 05-1) and we followed a strategy based on k-mer analysis. By breaking down the reads obtained by Illumina sequencing into short nucleotide sequences of defined length k (k-mers), several characteristics of genomes, like size, heterozygosity and repeat content, can be analyzed, that would normally require a complete *de novo* assembly. As basis for our analysis, DNA was generated from urediospores of the *P. pachyrhizi* isolate Brazil 05-1. A total of 47 Gb Illumina whole-genome sequencing data (100 bp paired-end reads) were then subjected to analysis using the program JELLYFISH (Marçais and Kingsford, 2011). In the 17-mer distribution depicted in **Figure 1**, two peaks could be differentiated at a depth of 37 and 75. This can be explained by the dikaryotic nature of the urediospores of rust fungi, which means that these organisms maintain two haploid nuclei separately during prolonged stages of their lifecycle. The two peaks in the k-mer histogram point to a high degree of heterozygosity between the two nuclei or to largely heterozygotic regions within the haploid nuclei. Similar results were also observed by Zheng et al. (2013) in the case of the wheat stripe rust fungus.

**FIGURE 1:**
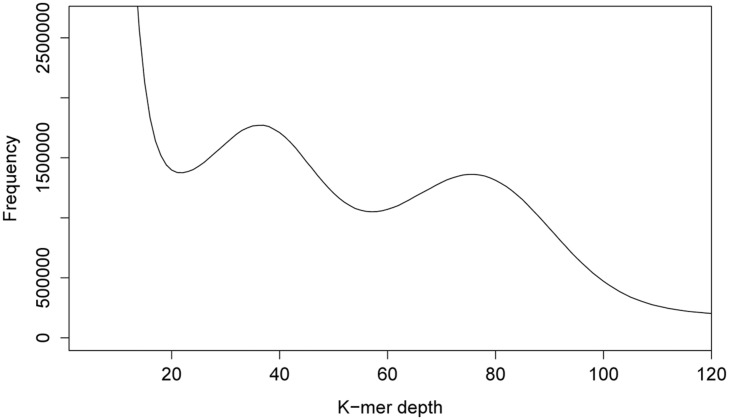
**K-mer analysis for *P. pachyrhizi* whole-genome sequencing data.** The 17-mer distribution for 47 Gb of 100 bp paired-end Illumina whole-genome sequencing data indicates two peaks at a depth of 37 and 75. These findings point to a possibly highly repetitive genome with a high degree of heterozygosity between the genomes of the two haploid nuclei.

By adding up the products of k-mer depth coverage and frequency for each pair of values in ****Figure 1****, divided by the depth coverage of the first peak (=37), the size of the genome in bp was computed, similarly as in (Li et al., 2010). Values, smaller than the first minimum in **Figure 1**, were considered noise caused by sequencing errors and were excluded from the calculation. Based on this analysis, the overall size of the dikaryotic genome of *P. pachyrhizi* is at most around 1 Gb. However, due to the unknown degree of heterozygosity between or within the genomes of both nuclei, this might be an overestimation (see above). The minimal size of the haploid genome can be estimated to be around 500 Mb, based on the second peak in the k-mer analysis (**Figure 1**). This would place the genome of the Asian soybean fungus in the same range as published rust genomes, e.g., *Hemileia vastatrix* (733.5 Mb) and *Uromyces* spp. (420 Mb; **Table 1**). It should be noted, however, that the analysis method might considerably influence the outcome of such genome size estimations. The genome size of *H. vastatrix*, e.g., was estimated by DNA-staining in combination with flow cytometry which itself is prone to errors but has the advantage of not being sequencing-dependent (Bainard et al., 2010). Phenomena related to the partial heterozygosity of the *P. pachyrhizi* genome are only detectable by assembly or k-mer analysis as described above. Since we did not use a large insert size sequencing approach for genome size estimation, we obtained a N50 value of 569 bp after assembly and scaffolding with SOAPdenovo. This allowed no prediction on gene number or length. In future studies a combined BAC- and third generation sequencing approach hopefully will increase the assembly quality to a point at which comprehensive gene predictions become possible.

Working with organisms, whose genome has been sequenced provides many advantages over working with non-sequenced species. Besides the comprehensive prediction of all genes, intra-genomic structural analyses or comparative genome analyses between different species become possible. An alternative to genomic-based approaches in large-scale analyses of plant-pathogen-interactions, however, is the use of transcriptomics, proteomics, or metabolomics (Tan et al., 2009). Up to now, only limited information is available on *P. pachyrhizi* transcriptomics, though very recent publications have broadened the view on particular aspects of the infection process of *P. pachyrhizi* (Tremblay et al., 2010, 2012, 2013; Link et al., 2013). For instance, Illumina-based transcriptome profiling at several stages of soybean leaf infection has led to the identification of nearly 19,000 transcripts not previously identified in other rust fungi (Tremblay et al., 2013). This would imply a much larger gene complement in the soybean rust than in other rust fungi. So far, the numbers of genes reported in rust fungi are between 15,000 and 20,000 genes (Duplessis et al., 2014). Although biases in the RNA-Seq approach can not be excluded, it is possible that the *P. pachyrhizi* genome has experienced a high level of gene duplication during its evolution along with important transposable element activity that could explain the huge genome size predicted for this species. There is an urgent need for genome sequences as prerequisite for accurate large scale expression analysis and more RNA-seq efforts are needed. Without a genome, transcript reads have to be assembled first and not only RNA quality and sequencing technique used will influence the resulting assembly quality but also the algorithms used for assembly. And even if these problems could be sufficiently solved, the resulting contigs are much smaller than transcribed ORFs, limiting for example predictions of putatively secreted proteins. Also, redundancy within gene families could be better resolved when compared to a reference genome sequence.

Hopefully in the near future, the development of novel sequencing and assembly strategies, together with dropping costs for NGS, will make the sequencing of large and complex genomes more affordable and will help to unravel the secrets of the genome of *P. pachyrhizi*.

## Conflict of Interest Statement

The authors declare that the research was conducted in the absence of any commercial or financial relationships that could be construed as a potential conflict of interest.

## Footnotes

1 http://genome.jgi.doe.gov/programs/fungi/1000fungalgenomes.jsf

2 http://genome.jgi.doe.gov/genome-projects/pages/project-status.jsf?projectId=16847
